# Ten-Day Quadruple Therapy Comprising Low-Dose Rabeprazole, Bismuth, Amoxicillin, and Tetracycline Is an Effective and Safe First-Line Treatment for Helicobacter pylori Infection in a Population with High Antibiotic Resistance: a Prospective, Multicenter, Randomized, Parallel-Controlled Clinical Trial in China

**DOI:** 10.1128/AAC.00432-18

**Published:** 2018-08-27

**Authors:** Yong Xie, Zhenhua Zhu, Jiangbin Wang, Lingxia Zhang, Zhenyu Zhang, Hong Lu, Zhirong Zeng, Shiyao Chen, Dongsheng Liu, Nonghua Lv

**Affiliations:** a The First Affiliated Hospital of Nanchang University, Nanchang, China; b China-Japan Union Hospital, Jilin University, Changchun, China; c Central Hospital of Xi'an City, Xi'an, China; d Nanjing No. 1 Hospital, Nanjing, China; e Renji Hospital, Shanghai Jiao Tong University School of Medicine, Shanghai, China; f The First Affiliated Hospital of Sun Yat-sen University, Guangzhou, China; g Zhongshan Hospital, Fudan University, Shanghai, China

**Keywords:** Helicobacter pylori, treatment, amoxicillin, tetracycline, safety, antimicrobial safety

## Abstract

The objective of this study was to investigate the efficacy and safety of 10-day bismuth quadruple therapy with amoxicillin, tetracycline, or clarithromycin and different doses of rabeprazole for first-line treatment of Helicobacter pylori infection. This multicenter, randomized, parallel-controlled clinical trial was conducted between March 2013 and August 2014. A total of 431 H. pylori-infected patients with duodenal ulcers were enrolled and randomized into four treatment groups (1:1:1:1) for 10 days, as follows: (i) a group receiving a low dose of rabeprazole of 10 mg twice a day (b.i.d.) (LR dose) plus bismuth, amoxicillin, and clarithromycin (LR-BAC); (ii) a group receiving LR plus bismuth, amoxicillin, and tetracycline (LR-BAT); (iii) a group receiving a high dose of rabeprazole of 20 mg b.i.d. (HR dose) plus bismuth, amoxicillin, and clarithromycin (HR-BAC); and (iv) a group receiving HR-BAT. Antimicrobial susceptibility was assessed by the Etest method. The primary outcome was H. pylori eradication at 4 weeks after the treatment. The per-protocol (PP) eradication rates in the LR-BAC, LR-BAT, HR-BAC, and HR-BAT groups were 94.1%, 91.9%, 94.8%, and 91.9%, respectively, while the intention-to-treat (ITT) eradication rates in those groups were 87.2%, 87.2%, 87.7%, and 86%, respectively. There was no significant difference between the four groups in PP analysis (*P* = 0.799) and ITT analysis (*P* = 0.985). The efficacies of four-treatment therapy were not affected by antibiotic resistance. The adverse events in the four treatment groups were similar; central nervous system (CNS) and gastrointestinal symptoms were the most common reported. Bismuth-containing quadruple therapy with low-dose rabeprazole, amoxicillin, and tetracycline is a good option for first-line treatment of H. pylori infection in a population with high antibiotic resistance. (This study is registered at Chinese Clinical Trials Registry [www.chictr.org.cn] under number ChiCTR1800014832.)

## Abstract (precis)

The objective of this study was to investigate the efficacy and safety of 10-day bismuth quadruple therapy with amoxicillin, tetracycline, or clarithromycin and different doses of rabeprazole for first-line treatment of Helicobacter pylori infection. This multicenter, randomized, parallel-controlled clinical trial was conducted between March 2013 and August 2014.

## INTRODUCTION

Helicobacter pylori is the major cause of chronic gastritis, peptic ulcers, gastric adenocarcinoma, gastric mucosa-associated lymphoid tissue (MALT) lymphoma, and various other digestive system diseases (1–3). Eradicating the pathogen is crucial for the prevention and treatment of these diseases (4). In China, resistance to commonly used antibiotics recommended for H. pylori eradication is increasing; for example, 20 to 50% of cases are resistant to clarithromycin (5, 6). With such high resistance rates, the eradication rate of the standard triple therapy for H. pylori infection has declined to <80%, which is unacceptable for clinical practice (7–9). The Maastricht V consensus recommends bismuth quadruple therapy as the first-line treatment protocol in regions with high rates of clarithromycin resistance (>15 to 20%) (10). In addition, tetracycline has been recommended as an anti-H. pylori drug worldwide as it is a cost-effective antibiotic with a low rate of resistance. Regarding the efficacy of quadruple therapy containing tetracycline and amoxicillin, results have been inconsistent. Some clinical studies have indicated that combining tetracycline and amoxicillin may reduce their eradication benefits. Nevertheless, all of these studies have some limitations, such as small sample size and lack of a control group, that compromise their reliability for assessing the value of that drug combination in H. pylori eradication therapy (11, 12).

Proton pump inhibitors (PPIs) play a critical role in H. pylori eradication protocols (13). However, different PPIs display various acid-suppressing effects and antibacterial activities. Rabeprazole is a type of PPI that is stable, highly efficient, and only slightly affected by the *CYP2C19* gene polymorphism. Its acid-suppressing effect is dose dependent. However, different dosages of this drug have been recommended for H. pylori eradication worldwide. High-dose rabeprazole (HR) (20 mg twice daily [b.i.d.]) is recommended in the Maastricht V consensus, whereas both low-dose rabeprazole (LR; 10 mg b.i.d.) and high-dose regimens are employed in China (10). Some studies have indicated that increasing the PPI dose in triple therapy containing omeprazole or lansoprazole can improve the eradication efficacy (14–16), whereas another study reported similar efficacies between low-dose and high-dose rabeprazole in triple-drug therapy (17). Thus, the appropriate dose of rabeprazole for eradication therapy remains controversial. Furthermore, it has not yet been reported whether increasing the dose of rabeprazole in quadruple-drug therapy can improve the efficacy of treatment. In this study, we investigate the efficacy and safety of a quadruple-drug therapy combining different dosages of rabeprazole, bismuth, amoxicillin, and tetracycline for H. pylori infection in China.

## RESULTS

### Demography.

**FIG 1:**
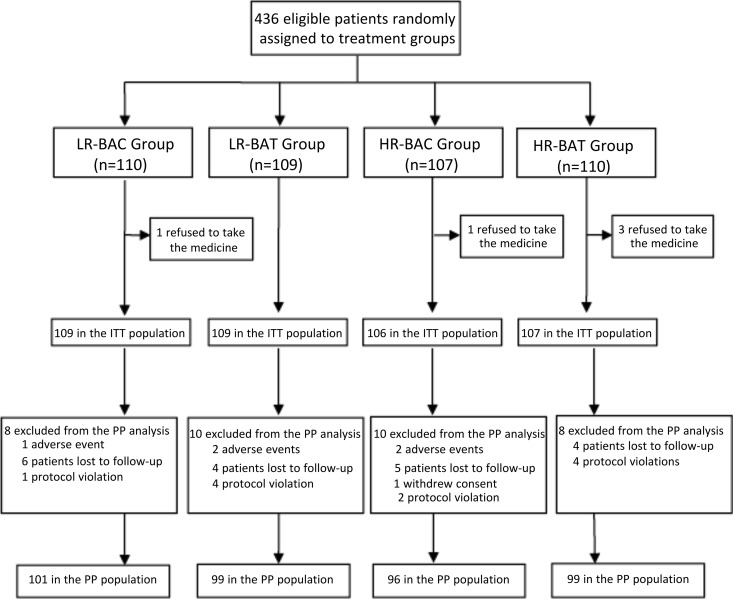
Flow diagram of this study. LR-BAC group, rabeprazole at 10 mg b.i.d. plus bismuth, amoxicillin, and clarithromycin; LR-BAT group, rabeprazole at 10 mg b.i.d plus bismuth, amoxicillin, and tetracycline; HR-BAC group, rabeprazole at 20 mg b.i.d. plus bismuth, amoxicillin, and clarithromycin; HR-BAT group, rabeprazole at 10 mg b.i.d plus bismuth, amoxicillin, and tetracycline. ITT, intention-to-treat; PP, per-protocol.

A total of 436 eligible patients were assigned randomly to four groups (a group of 110 cases receiving LR plus bismuth, amoxicillin, and clarithromycin [LR-BAC],; a group of 109 cases receiving LR plus bismuth, amoxicillin, and tetracycline [LR-BAT], a group of 107 cases receiving HR plus bismuth, amoxicillin, and clarithromycin [HR-BAC],; and a group of 110 cases receiving HR-BAT). Of these 436 patients, 5 patients were excluded from the intention-to-treat (ITT) population as they refused to take the study drugs. Thus, 431 patients received the randomized drug treatment and were enrolled in the ITT population (109 patients in the LR-BAC group, 109 in the LR-BAT group, 106 in the HR-BAC group, and 107 in the HR-BAT group). During the study, a total of 36 patients (8.4%) were excluded from the per-protocol (PP) analysis: 8 in the LR-BAC group (7.3%), 10 in the LR-BAT group (9.2%), 10 in the HR-BAC group (9.4%), and 8 in the HR-BAT group (7.5%). They were excluded primarily because of poor compliance, withdrawal of consent, or loss to follow-up (Fig. 1). The demographic and baseline characteristics of the patients involved in the ITT analysis are shown in Table 1.

**TABLE 1:** Baseline characteristics

Parameter	Value for the group^*a*^				*P*
	LR-BAC (*n* = 109)	LR-BAT (*n* = 109)	HR-BAC (*n* = 106)	HR-BAT (*n* = 107)	
Gender					
Male	64	75	67	64	0.4123
Female	45	34	39	43	
Age (yr [range])	38.9 ± 12.01 (20–65)	41.8 ± 13.31 (18–70)	38.8 ± 12.81 (18–69)	41.4 ± 13.35 (18–69)	0.1895
Smoking status					
Former	1 (0.9)	2 (1.8)	0	0	0.2980
Current	31 (28.4)	39 (35.8)	34 (32.1)	43 (40.2)	0.3021
Alcohol use					0.2572
Never	66 (60.6)	68 (62.4)	61 (57.5)	68 (63.6)	
Sometimes	40 (36.7)	39 (35.8)	36 (34.0)	32 (29.9)	
Often	3 (2.8)	2 (1.8)	9 (8.5)	7 (6.5)	
Drug use history					
Clarithromycin	5 (4.6)	2 (1.8)	0	2 (1.9)	0.1300
Amoxicillin	47 (43.1)	45 (41.3)	45 (42.5)	45 (42.1)	0.9943
Tetracycline	1 (0.9)	4 (3.7)	5 (4.7)	0	0.0703
Bismuth	2 (1.8)	5 (4.6)	5 (4.7)	4 (3.7)	0.6576
Peptic ulcer history	7 (6.4)	14 (12.8)	5 (4.7)	9 (8.4)	0.1486
Upper gastrointestinal bleeding history	1 (0.9)	4 (3.7)	3 (2.8)	1 (0.9)	0.3846
Chronic gastritis history	12 (11.0)	13 (11.9)	10 (9.4)	7 (6.5)	0.5626
Family history of gastric cancer	2 (1.8)	1 (0.9)	0	4 (3.7)	0.1636

a Unless otherwise stated, values are number of patients (percentage of patients). LR-BAC group, rabeprazole at 10 mg b.i.d. plus bismuth, amoxicillin, and clarithromycin; LR-BAT group, rabeprazole at 10 mg b.i.d. plus bismuth, amoxicillin, and tetracycline; HR-BAC group, rabeprazole at 20 mg b.i.d. plus bismuth, amoxicillin, and clarithromycin; HR-BAT group, rabeprazole at 10 mg b.i.d. plus bismuth, amoxicillin, and tetracycline.

### H. pylori eradication rate.

Table 2 displays the major outcomes of eradication therapies. PP analysis showed that the overall eradication rate was 93.2%, and the H. pylori eradication rate of each treatment group was as follows: 94.1% in the LR-BAC group, 91.9% in the LR-BAT group, 94.8% in the HR-BAC group, and 91.9% in the HR-BAT group (Table 2). ITT analysis showed that the overall eradication rate was 87%, and the H. pylori eradication rate of each treatment group was as follows: 87.2% in the LR-BAC group, 87.2% in the LR-BAT group, 87.7% in the HR-BAC group, and 86% in the HR-BAT group (Table 2). The four therapies had comparable eradication rates (*P* = 0.7990 in PP analysis and *P* = 0.9850 in ITT analysis).

**TABLE 2:** H. pylori eradication rate of each group in PP and ITT analysis^*a*^

Population	Eradication by group (%)^*b*^								*P*
	LR-BAC		LR-BAT		HR-BAC		HR-BAT		
	Rate^*c*^	95% CI	Rate	95% CI	Rate	95% CI	Rate	95% CI	
PP	94.1 (95/101)	87.5–97.8	91.9 (91/99)	84.7–96.4	94.8 (91/96)	88.3–98.3	91.9 (91/99)	84.7–96.4	0.7990
ITT	87.2 (95/109)	79.4–92.8	87.2 (95/109)	79.4–92.8	87.7 (93/106)	79.9–93.3	86.0 (92/107)	77.9–91.9	0.9850

a PP, per protocol; ITT, intention to treat.

b LR-BAC group, rabeprazole at 10 mg b.i.d. plus bismuth, amoxicillin, and clarithromycin; LR-BAT group, rabeprazole at 10 mg b.i.d. plus bismuth, amoxicillin, and tetracycline; HR-BAC group, rabeprazole at 20 mg b.i.d. plus bismuth, amoxicillin, and clarithromycin; HR-BAT group, rabeprazole at 10 mg b.i.d. plus bismuth, amoxicillin, and tetracycline.

c Values in parentheses are the number of patients with a negative result on the urea breath test/total number of patients.

### Adverse events and compliance.

Patients who received at least one dose of eradication drugs were included in the adverse-event analysis. The frequencies of adverse events are summarized in Table 3. A total of 57 adverse events were recorded in 33 of 431 (7.7%) patients, representing 8 of 109 (7.3%) in the LR-BAC therapy, 5 of 109 (4.6%) in the LR-BAT therapy, 13 of 106 (12.3%) in the HR-BAC therapy, and 7 of 107 (6.5%) patients in the HR-BAT therapy. Central nervous system (CNS) symptoms, including dizziness, headache, and dysgeusia, and gastrointestinal symptoms were the most common reported adverse events. All of the adverse events were graded as mild. The differences in the overall incidence of gastrointestinal disorders among the four groups were statistically significant (*P* = 0.0370); the incidence of gastrointestinal disorders was highest in the HR-BAC group and lowest in the LR-BAT group.

**TABLE 3:** Adverse events of each group

AE category^*a*^	Frequency by group (no. of patients [%])^*b*^				*P*
	LR-BAC (*n* = 109)	LR-BAT (*n* = 109)	HR-BAC (*n* = 106)	HR-BAT (*n* = 107)	
Total no. of AEs	10	6	30	11	
No. of patients with AEs	8 (7.3)	5 (4.6)	13 (12.3)	7 (6.5)	0.1840
Liver system	0	0	2 (1.9)	0	
CNS disorders	6 (5.5)	2 (1.8)	10 (9.4)	4 (3.7)	0.0733
Headache	0	0	3 (2.8)	1 (0.9)	
Dizziness	3 (2.8)	2 (1.8)	6 (5.7)	3 (2.8)	0.4220
Dysgeusia	3 (2.8)	0	4 (3.8)	0	
Skin rash	0	2 (1.8)	1 (0.9)	3 (2.8)	
Gastrointestinal disorders	3 (2.8)	1 (0.9)	7 (6.6)	1 (0.9)	0.0370
Vomiting	1 (0.9)	0	2 (1.9)	0	
Abdominal pain	0	0	2 (1.9)	0	
Bloating	2 (1.8)	1 (0.9)	3 (2.8)	0	
diarrhea	0	0	0	1 (0.9)	
Other	1 (0.9)	1 (0.9)	4 (3.8)	1 (0.9)	

a AE, adverse event.

b Total AE values are the numbers of events. LR-BAC group, rabeprazole at 10 mg b.i.d. plus bismuth, amoxicillin, and clarithromycin; LR-BAT group, rabeprazole at 10 mg b.i.d. plus bismuth, amoxicillin, and tetracycline; HR-BAC group, rabeprazole at 20 mg b.i.d. plus bismuth, amoxicillin, and clarithromycin; HR-BAT group, rabeprazole at 10 mg b.i.d. plus bismuth, amoxicillin, and tetracycline.

The overall compliance rate of the four groups was 98.1% (423/431), while the compliance rates in the LR-BAC, LR-BAT, HR-BAC, and HR-BAT groups were 99.1% (108/109), 97.2% (106/109), 97.2% (103/106), and 99.1% (106/107), respectively.

### Impacts of antibiotic resistances on eradication rates.

H. pylori strains were successfully isolated from 288 patients. Drug sensitivity testing was undertaken at baseline, and the rates of resistant strains to amoxicillin, clarithromycin, and tetracycline were 4.5% (13/288), 18.4% (53/288), and 0.7% (2/288), respectively. Table 4 shows no differences in rates of resistance to amoxicillin, clarithromycin, and tetracycline among four treatment groups in the ITT and PP populations (*P* > 0.05).

**TABLE 4:** Antibiotic resistance analysis of each group

Drug and population	Resistance rate by group^*a*^				*P*
	LR-BAC	LR-BAT	HR-BAC	HR-BAT	
Amoxicillin					
ITT	3/73 (4.1)	3/71 (4.2)	1/73 (1.4)	6/71 (8.5)	0.2339
PP	2/68 (2.9)	2/63 (3.2)	1/66 (1.5)	5/64 (7.8)	0.3723
Clarithromycin					
ITT	15/73 (20.5)	17/71 (23.9)	6/73 (8.2)	15/71 (21.1)	0.0698
PP	14/68 (20.6)	14/63 (22.2)	6/66 (9.0)	14/64 (21.9)	0.0752
Tetracycline					
ITT	0/73 (0)	0/71 (0)	1/73 (1.4)	1/71 (1.4)	
PP	0/68 (0)	0/63 (0)	1/66 (1.5)	1/64 (1.6)	

a Values are the number of resistant strains isolated/total number of strains isolated (percentage). LR-BAC group, rabeprazole at 10 mg b.i.d. plus bismuth, amoxicillin, and clarithromycin; LR-BAT group, rabeprazole at 10 mg b.i.d. plus bismuth, amoxicillin, and tetracycline; HR-BAC group, rabeprazole at 20 mg b.i.d. plus bismuth, amoxicillin, and clarithromycin; HR-BAT group, rabeprazole at 10 mg b.i.d. plus bismuth, amoxicillin, and tetracycline.

Table 5 shows the impacts of antibiotic resistance on the eradication rates of each therapy in the PP population. No differences in eradication rates existed between clarithromycin-resistant and -sensitive strains and between amoxicillin-resistant and -sensitive strains in the four treatment groups. In the LR-BAC group, although patients with isolated clarithromycin susceptibility achieved a higher cure rate than those with isolated clarithromycin resistance (96.3% versus 85.7%), the differences failed to reach statistical significance (*P* = 0.185). The eradication rate of strains dually resistant to amoxicillin and clarithromycin was 100% (7/7).

**TABLE 5:** Impact of antibiotic resistance on the eradication rate in the PP population^*a*^

Group (*n*)^*b*^	Eradication rate by resistance type^*c*^										
	Amoxicillin			Clarithromycin			Tetracycline		Dual		
	Yes	No	*P*	Yes	No	*P*	Yes	No	Yes	No	*P*
LR-BAC (68)	2/2 (100)	62/66 (93.9)	1.000	12/14 (85.7)	52/54 (96.3)	0.185	0	64/68 (94.1)	1 (100)	63/67 (94.0)	
LR-BAT (63)	2/2 (100)	54/61 (88.5)	1.000	14/14 (100)	42/49 (85.7)	0.333	0	56/63 (88.9)	2/2 (100)	54/61 (88.5)	1.000
HR-BAC (66)	1/1 (100)	61/65 (93.8)		6/6 (100)	56/60 (93.3)	1.000	1/1 (100)	61/65 (93.8)	1/1 (100)	61/65 (93.8)	
HR-BAT (64)	5/5 (100)	55/59 (93.2)	1.000	14/14 (100)	46/50 (92.0)	0.568	1/1 (100)	59/63 (93.7)	3 (100)	57/61 (93.4)	1.000

a PP, per protocol.

b LR-BAC group, rabeprazole at 10 mg b.i.d. plus bismuth, amoxicillin, and clarithromycin; LR-BAT group, rabeprazole at 10 mg b.i.d. plus bismuth, amoxicillin, and tetracycline; HR-BAC group, rabeprazole at 20 mg b.i.d. plus bismuth, amoxicillin, and clarithromycin; HR-BAT group, rabeprazole at 10 mg b.i.d. plus bismuth, amoxicillin, and tetracycline.

c Values are the number of eradications/total number of patients (percentage).

## DISCUSSION

This study performed the first multicenter, randomized, controlled trial to test whether 10-day quadruple therapy comprising low-dose rabeprazole, bismuth, amoxicillin, and tetracycline is an effective and safe first-line treatment for H. pylori infection in areas with a high level of clarithromycin resistance. Both ITT and PP analyses showed that four therapies cured most patients with H. pylori infection (87.2%, 87.2%, 87.7%, and 86% for the ITT population and 94.1%, 91.9%, 94.8%, and 91.9% for the PP population for the LR-BAC, LR-BAT, HR-BAC, and HR-BAT groups, respectively). These results were similar to the efficacy (ITT, 88.8%; PP, 94.9%) of a 14-day quadruple-therapy regimen (including lansoprazole, bismuth, amoxicillin, and clarithromycin), as reported recently in China (18). The eradication rates of all the groups in the ITT population were higher than 85%, indicating the acceptability of these eradication regimens. The resistance rates of H. pylori to tetracycline and amoxicillin were low in many regions (6), but the combination of these two drugs is not recommended according to the current consensus, which may be partially due to their mutual pharmacological antagonism; the results of this study, however, suggest that the combination achieves a high eradication rate. In addition, more than 95% of patients were compliant with the study medication in the four therapy groups. Therefore, the results indicate that 10-day quadruple therapy comprising tetracycline and amoxicillin can be recommended for the first-line treatment of H. pylori infection in the areas of high clarithromycin resistance.

Recent studies have revealed that increasing the dose of PPIs can increase the H. pylori eradication rate (19, 20), which may be associated with the following factors: (i) a high dose of PPI can raise the pH of the stomach and efficiently inhibit the growth of H. pylori; (ii) some antibiotics used for H. pylori eradication therapy, such as amoxicillin, clarithromycin, tetracycline, and levofloxacin, are pH dependent in that their antibacterial activity is significantly affected by a low-pH environment; (iii) a high dose of PPI can improve the concentrations of antibiotics in gastric mucus. Rabeprazole, a second-generation PPI, is a benzimidazole-based compound, characterized by a large dissociation constant and rapid onset. Hence, it can increase the pH value in the stomach within a short time and strengthen the effects of antibiotics in an acidic environment, and its acid-suppressing effect is dose dependent. The Maastricht V consensus recommends a dose of 10 to 20 mg b.i.d. for H. pylori eradication therapy (10). A national multicenter study in Japan demonstrated that low-dose rabeprazole (10 mg b.i.d.) was likely to achieve efficacy similar to that of a higher dose (20 mg b.i.d.) in a rabeprazole-based triple-therapy regimen (17). Our previous study concerning a low-dose rabeprazole-based quadruple-therapy regimen (bismuth plus amoxicillin plus furazolidone) also reported a high eradication rate (PP, 92.3%; ITT, 86.1%) (21). Nevertheless, whether high-dose rabeprazole achieves a higher eradication rate and similar adverse events in a quadruple-therapy regimen has not yet been reported. In this study, we found that a quadruple regimen containing low-dose rabeprazole (LR-BAC and LR-BAT groups) was able to achieve efficacy similar to that of a regimen with a high dose (PP, >90%; ITT, >85%). Therefore, quadruple-therapy eradication regimens containing low-dose rabeprazole are applicable for H. pylori eradication.

Antibiotic resistance is an important factor for the failure of H. pylori eradication. A national multicenter study in Japan showed that the eradication rate of the standard triple-therapy eradication regimen containing clarithromycin was 95% (360/379) for clarithromycin-susceptible strains but only 50% (30/60) for clarithromycin-resistant strains, which suggested that the eradication regimen containing clarithromycin had substantially compromised efficacy against clarithromycin-resistant strains (22). Our results revealed that 18.4% (53/288), 4.5% (13/288), and 0.7% (2/288) of the patients were resistant to clarithromycin, amoxicillin, and tetracycline, respectively. The Maastricht V consensus recommends bismuth quadruple therapy as the first-line treatment protocol in regions with high rates of clarithromycin resistance (>15 to 20%) (10). Ideally, clarithromycin should be avoided, and a combination of alternative antibiotics for which resistance is low (e.g., amoxicillin, tetracycline, and furazolidone) should be used. Clarithromycin resistance was about 20% in each treatment group of our study. In the LR-BAC group, although clarithromycin resistance did not affect the efficacy significantly, there was trend toward a lower eradication rate (96.3% cure rate for clarithromycin-susceptible strains and 85.7% for clarithromycin-resistant strains). However, the eradication rate was high and not influenced by clarithromycin resistance in the LR-BAT and HR-BAT groups (100% cure rate for clarithromycin-resistant strains). It is not surprising that these two therapies have high eradication rates as they do not contain clarithromycin, and the rates of resistance to amoxicillin and tetracycline are low. Interestingly, HR-BAC therapy also achieved a 100% (14/14) cure rate for clarithromycin-resistant strains, which suggests that high-dose rabeprazole helps to overcome clarithromycin resistance, but more clarithromycin-resistant strains are needed to verify this possibility. A 100% (7/7) cure rate was achieved in patients with dual resistance to amoxicillin and clarithromycin. The high eradication efficacy for H. pylori strains with dual antibiotic resistance might be owing to the combined use of tetracycline and bismuth and to the long treatment duration of amoxicillin; in addition, the small sample size of H. pylori strains with dual antibiotic resistance is one of the important reasons. Although the resistance rate of amoxicillin in our study was slightly higher than that of other previous studies (6, 10), the resistance rate was still low (4.5% resistance). In addition, tetracycline resistance (0.7%; 2/288) is very low in our study, and dual resistance to amoxicillin and tetracycline was zero in each treatment group. The high eradication rate of the treatment for amoxicillin-resistant strains in our study might have resulted from the combined use of tetracycline and bismuth and from the long treatment duration. Therefore, bismuth-containing quadruple therapies with tetracycline may be a good treatment choice in areas of low tetracycline resistance.

Our study showed that the treatment was well tolerated. In our study, the incidence of adverse events was 7.7% (33/431) in the overall population, which was similar to the incidence in a 10-day furazolidone-based quadruple regimen with low-dose rabeprazole (9.4%, 17/180) (21). CNS symptoms, including dizziness, headache, and dysgeusia, and gastrointestinal symptoms were the most common reported adverse events, most of which were mild even without treatment. The incidence of adverse events was highest in the HR-BAC group (12.3%, 13/106) and lowest in the LR-BAT group (4.6%, 5/109), which indicates that the quadruple regimen containing tetracycline and low-dose rabeprazole is the safest of the four therapies that we tested. In this study, no mortality was recorded. Therefore, the safety of these four therapies was well accepted.

The advantages of this study include comparisons with randomized controlled trials and a multicenter study covering the east, west, north, south, and central regions of China (seven tertiary hospitals). Additionally, this study determined the impact of antibiotic resistance on eradication results. However, there are several limitations of this study. First, the H. pylori culture was positive in just about 68% of the cases. Second, the sample size of resistant strains was small for analyzing the impact of antibiotic resistance on the eradication rate. Third, there was no long-term follow-up on the impact on gut microbiota, which is the research focus at present.

In conclusion, a high H. pylori eradication rate could be achieved using quadruple therapy with a combination of rabeprazole, bismuth, amoxicillin, and tetracycline in areas of high clarithromycin resistance. In addition, low-dose rabeprazole shows efficacy comparable to that of a high dose while having fewer adverse events. Therefore, 10-day quadruple therapy consisting of low-dose rabeprazole, bismuth, amoxicillin, and tetracycline can be recommended for first-line empirical treatment of H. pylori infection in China, especially in areas of high clarithromycin resistance.

## MATERIALS AND METHODS

### Study design.

This study was a multicenter, randomized, parallel-controlled study conducted at seven tertiary hospitals in China from March 2013 to October 2014, including the First Affiliated Hospital of Nanchang University, Zhongshan Hospital Affiliated to Fudan University, the First Affiliated Hospital of Sun Yat-sen University, China-Japan Union Hospital of Jilin University, Nanjing No. 1 Hospital, Shanghai Renji Hospital, and the Central Hospital of Xi'an City. This study was approved by the Ethics Committee of the First Affiliated Hospital of Nanchang University. Consecutive H. pylori-infected adult patients (≥18 years old) with active duodenal ulcers were recruited for this study. Each patient signed an informed consent. This study is registered at the Chinese Clinical Trials Registry (www.chictr.org.cn) under clinical trial number ChiCTR1800014832.

### Eligibility criteria.

Consecutive H. pylori-infected patients with duodenal ulcers were prospectively recruited for this study. H. pylori infections were diagnosed by the [^13^C]urea breath test. Exclusion criteria included the use of antibiotics and bismuth in the preceding 4 weeks or histamine 2 (H2) receptor agonists and PPIs in the preceding 2 weeks, pregnancy or lactation at the time of the study, presence of clinically significant associated conditions (hepatic, cardio-respiratory or renal disease; neoplastic disease; coagulopathy), allergy to any of the medications in the regimens (especially penicillin allergy), and previous gastric surgery.

### Interventions.

The eligible patients were randomly assigned in a 1:1:1:1 ratio to receive one of the following four regimens for 10 days: LR-BAC, LR-BAT, HR-BAC, or HR-BAT. Randomization was conducted by a computer-generated random number sequence, and the assignments were sealed in opaque envelopes. This study was an open-label trial, and both the physicians and patients were aware of the treatment received.

The four regimens were as follows: LR-BAC (10 mg of rabeprazole and 220 mg of bismuth b.i.d., plus 1,000 mg of amoxicillin and 500 mg of clarithromycin b.i.d.), LR-BAT (10 mg of rabeprazole and 220 mg of bismuth b.i.d., plus 1,000 mg of amoxicillin and 750 mg of tetracycline b.i.d.), HR-BAC (20 mg of rabeprazole and 220 mg of bismuth b.i.d., plus 1,000 mg of amoxicillin and 500 mg of clarithromycin b.i.d.), and HR-BAT (20 mg of rabeprazole and 220 mg of bismuth b.i.d., plus 1,000 mg of amoxicillin and 750 mg of tetracycline b.i.d.).

The drugs and the manufacturers were as follows: rabeprazole (Pariet; China Eisai, Shuzhou, China), amoxicillin (Zhuhai United Laboratories, Zhuhai, China), clarithromycin (Klacid; Shanghai Abbott Laboratories, Shanghai, China), bismuth potassium citrate (Livzon Dele; Livzon Pharmaceutical Group, Zhuhai, China), and tetracycline (Guangdong Huanan Pharmaceutical Group, Dongguan, China).

In this study, rabeprazole and bismuth potassium citrate were given 30 min before the morning and evening meals. Antibiotics were given 30 min after the morning and evening meals. The patients kept a diary to record any side effects or discomforts that occurred during the 10-day treatment period. After H. pylori eradication therapy, all of the patients were administered 10 mg of rabeprazole once a day (q.d.) for 11 days. A staff member who was blinded to the eradication performed the urea breath tests 4 weeks after completion of therapy. Eradication was defined as a negative result on the urea breath test.

### Culture and antimicrobial resistance.

Two biopsy specimens were harvested from the gastric antrum and corpus for H. pylori culture and antibiotic susceptibility testing (23, 24). Antibiotic susceptibility was assessed using the Etest method (bioMérieux, Marcy l'Etoile, France). H. pylori subculturing was done by rubbing the specimens on the surface of a Campylobacter agar (Oxoid, Basingstoke, United Kingdom) supplemented with 5% sheep's blood (Bio-kont, Zhejiang, China) containing vancomycin, trimethoprim, polymyxin B, and amphotericin B (Duly Biotech, Nanjing, China), followed by incubation at 37°C under microaerobic conditions (5% O_2_, 10% CO_2_, and 85% N_2_) for 3 to 5 days. H. pylori strains were tested for tetracycline, clarithromycin, and amoxicillin susceptibility using the Etest (bioMérieux, Marcy l'Etoile, France). H. pylori strain ATCC 43504 was included as an antibiotic susceptibility testing quality control. All antibiotic susceptibility tests were conducted at the Institute of Gastroenterology and Hepatology, First Affiliated Hospital of Nanchang University. Tetracycline resistance was defined as a MIC of >2 μg/ml against H. pylori, while clarithromycin resistance and amoxicillin resistance MICs were >1 μg/ml and >1 μg/ml, respectively (25).

### Outcomes.

The primary outcome of the study was the eradication rate of H. pylori in each treatment group. The secondary outcomes were the frequency of adverse events, the compliance rate, the antibiotic resistance rates, and the effect of antibiotic resistance on the eradication rate in each group.

### Sample size estimation and statistical analysis.

The necessary sample size was calculated using the software nQuery. Assuming an H. pylori eradication rate of 90% for each treatment group, an equivalence cutoff of 15%, a one-sided significance level of 2.5%, and power of at least 90%, each group requires at least 88 patients. Assuming that 20% of subjects would be lost to follow-up, we planned to recruit at least 440 participants (110 subjects per group) for the study.

Statistical analyses were performed using the software SAS, version 9.2. All the statistical tests were two-sided with a cutoff alpha (α) of 0.05, and the two-sided 95% confidence intervals (CIs) were calculated. The count variables were analyzed using the χ^2^ test, Fisher's exact test, or a multiple-group rank sum test, whereas the measurement data were evaluated by one-way analysis of variance (ANOVA) or a multiple-group rank sum test. A *P* value of <0.05 was considered statistically significant.
